# Effect on neonatal sepsis following immediate kangaroo mother care in a newborn intensive care unit: a post-hoc analysis of a multicentre, open-label, randomised controlled trial

**DOI:** 10.1016/j.eclinm.2023.102006

**Published:** 2023-05-18

**Authors:** Sugandha Arya, Suhail Chhabra, Richa Singhal, Archana Kumari, Nitya Wadhwa, Pratima Anand, Helga Naburi, Kondwani Kawaza, Sam Newton, Ebunoluwa Adejuyigbe, Bjorn Westrup, Nils Bergman, Siren Rettedal, Agnes Linner, Rahul Chauhan, Nisha Rani, Nicole Minckas, Sachiyo Yoshida, Suman Rao, Harish Chellani

**Affiliations:** a Department of Paediatrics, Vardhman Mahavir Medical College and Safdarjung Hospital, Ansari Nagar, New Delhi, 110029, India; b Translational Health Science and Technology Institute, NCR Biotech Science Cluster, 3rd Milestone, Faridabad-Gurgaon Expressway, Post Box #04, Faridabad, Haryana, 121001, India; c Department of Paediatrics and Child Health, School of Medicine, Muhimbili University of Health and Allied Sciences, Dar es Salaam, 255, Tanzania; d Department of Paediatrics and Child Health, Kamuzu University of Health Sciences, Blantyre, Malawi; e School of Public Health, Kwame Nkrumah University of Science and Technology, Accra Road, Kumasi, Ghana; f Department of Paediatrics and Child Health, Obafemi Awolowo University, Ile-Ife, Nigeria; g Department of Women's and Children's Health, Karolinska Institutet, Sweden; h Department of Paediatrics, Stavanger University Hospital, Norway; i Department of Clinical Science, Intervention and Technology, Karolinska Institutet, Stockholm, Sweden; j Department of Maternal, Newborn, Child and Adolescent Health and Ageing, World Health Organization, Switzerland; k Department of Neonatology, St John's Medical College Hospital, Bengaluru, India

**Keywords:** Immediate kangaroo mother care (iKMC), Low birth weight (LBW), Mother newborn care unit (MNCU), Neonatal sepsis

## Abstract

**Background:**

To implement the immediate Kangaroo mother care (iKMC) intervention in the previous multicentre, open-label, randomised controlled trial, the mother or a surrogate caregiver and neonate needed to be together continuously, which led to the concept of the Mother–Newborn Care Unit (MNCU). Health-care providers and administrators were concerned of the potential increase in infections caused by the continuous presence of mothers or surrogates in the MNCU. We aimed to assess the incidence of neonatal sepsis in sub-groups and the bacterial profile among intervention and control neonates in the study population.

**Methods:**

This is a post-hoc analysis of the previous iKMC trial, which was conducted in five level 2 Newborn Intensive Care Units (NICUs) one each in Ghana, India, Malawi, Nigeria, and Tanzania, in neonates with birth weight 1 to <1.8 kg. The intervention was KMC initiated immediately after birth and continued until discharge and compared to conventional care with KMC initiated after meeting stability criteria. The primary outcomes of this report were the incidence of neonatal sepsis in sub-groups, sepsis-related mortality and bacterial profile of isolates during hospital stay. The original trial is registered with the Australia and New Zealand Clinical Trials Registry (ACTRN12618001880235) and the Clinical Trials Registry-India (CTRI/2018/08/01536).

**Findings:**

Between November 30, 2017, and January 20, 2020, 1609 newborns in the intervention group and in the control group 1602 newborns were enrolled in iKMC study. 1575 newborns in the intervention group and 1561 in the control group were clinically evaluated for sepsis. Suspected sepsis was 14% lower in intervention group in sub-group of neonates with birth weight 1.0-<1.5 kg; RR 0.86 (CI 0.75, 0.99). Among neonates with birth weight 1.5-<1.8 kg, suspected sepsis was reduced by 24%; RR 0.76 (CI 0.62, 0.93). Suspected sepsis rates were lower in intervention group than in the control group across all sites. Sepsis related mortality was 37% less in intervention group than the control group; RR 0.63 (CI 0.47–0.85) which was statistically significant. The intervention group had fewer cases of Gram-negative isolates (n = 9) than Gram positive isolates (n = 16). The control group had more cases of Gram-negative isolates (n = 18) than Gram positive (n = 12).

**Interpretation:**

Immediate Kangaroo Mother care is an effective intervention to prevent neonatal sepsis and sepsis related mortality.

**Funding:**

The original trial was funded by the 10.13039/100000865Bill and Melinda Gates Foundation through a grant to the 10.13039/100004423World Health Organization (grant No. OPP1151718).

**Research in context**

**Evidence before this study**

Immediate Kangaroo Mother Care (iKMC) stands for immediate and continuous KMC, which means KMC to be started soon after birth (within 2 h) and to be given continuously (aiming up to 20 h per day) before and after stabilization. It is an evidence-based intervention to improve survival of low birth weight neonates. To implement intervention of iKMC, the mother and neonate need to be together continuously, which led to the concept of “Mother–Newborn Care Unit (MNCU)”. Healthcare providers and administrators are concerned of a potential increase in infections by the presence of mothers and surrogates in the MNCU. We searched PubMed for studies published by December, 2022, with the search terms “Neonatal sepsis,” or “neonatal infections,” and “kangaroo mother care” [MeSH], or “care method, kangaroo mother” [MeSH], or “skin to-skin contact” [MeSH], or “skin-to-skin care” [MeSH]. Kangaroo Mother Care – Cochrane review 2016 showed that KMC when started after LBW newborn has stabilized, reduces neonatal sepsis by 55%. We found no peer-reviewed studies which analysed the impact of KMC provision in Newborn Intensive Care Unit (NICU) on neonatal sepsis. However, the primary iKMC study results showed that, suspected sepsis was 18% lower in the intervention group than in the control group; RR 0.82 (CI 0.73–0.93).

**Added value of this study**

The aim of this study was post-hoc analyses of the effects of immediate and continuous KMC on incidence of neonatal sepsis. Sepsis related mortality was 37% less in intervention group than the control group, RR 0.63 (CI 0.47–0.85) which was statistically significant. The intervention group had statistically significant less incidence of sepsis across the categories of birth weight. Suspected sepsis rates were lower in intervention group than in the control group across all sites. Profile of bacterial isolates is different in Immediate KMC and control group.

**Implications of all the available evidence**

Immediate Kangaroo Mother Care is an effective intervention to prevent neonatal sepsis and sepsis related mortality.

## Introduction

A systematic review done by Cochrane research network published in 2016 showed that when Kangaroo Mother Care (KMC) is initiated after stabilization, there is a 40% decrease in neonatal mortality.^1^ However, evidence of the effect of initiating KMC immediately after birth without waiting for babies to stabilize was not available. To answer this research question, a multi-country randomized controlled trial was conducted in five countries (India, Ghana, Tanzania, Nigeria and Malawi). The immediate KMC (iKMC) trial was conducted from December 2017–January 2020 and was co-ordinated by the World Health Organization (WHO).^2^ In total 3211 neonates with birth weight 1 to <1.8 kg were enrolled. Intervention neonates received iKMC by 1 h of life and control neonates received conventional care in an incubator or a radiant warmer till they stabilized and were shifted to KMC ward thereafter. The primary outcomes of the iKMC trial were death in the neonatal period (the first 28 days of life) and in the first 72 h of life. Primary finding of iKMC trial was that intervention reduces relative risk of neonatal mortality by 25%; RR 0.75 (95% CI 0.64–0.89).^2^

iKMC stands for immediate and continuous KMC, which means KMC to be started soon after birth (within 2 h) and to be given continuously (aiming up to 20 h per day) before and after stabilization. To implement this intervention, the mother and neonate need to be together continuously, which led to the concept of “Mother–Newborn Care Unit (MNCU)”.

MNCU is a facility where sick and small neonates are cared for with their mothers, with all facilities of level 2 newborn care and provision for postnatal care to mothers (Mother-Newborn Couplet Care).^3^ The mother has her bed inside the Newborn Intensive Care Unit (NICU) and stays there day and night unlike conventional NICUs where mothers visit intermittently. When mother was not available, iKMC was provided by another person (surrogate) designated by the parents. Healthcare providers and administrators were concerned of a potential increase in infections by the presence of mothers and surrogates in the MNCU. However, the iKMC study results showed that, suspected sepsis was 18% lower in the intervention group than in the control group; RR 0.82 (CI 0.73–0.93).^2^

The objective of this study was post-hoc analysis of the effects of immediate and continuous KMC on incidence of neonatal sepsis across different weight categories and study sites. Another objective was to evaluate profile of causative pathogens and multidrug resistance patterns and sepsis related mortality.

## Methods

### Study design

The study protocol details including sample size calculation, recruitment procedure and intervention of primary iKMC study have been published.^4^ This multi-country, randomised controlled clinical trial was registered in Australian New Zealand Clinical Trials Registry number, ACTRN12618001880235; Clinical Trials Registry-India number, CTRI/2018/08/015369.) The study protocol was approved by the WHO Ethics Review Committee with reference number: EC0002901 and by local institutional review boards of the sites. Results of primary iKMC study have also been published.^2^ CONSORT reporting guidelines for randomised trials have been followed. This article presents post-hoc analysis of incidence of neonatal sepsis in sub-groups, sepsis related mortality and the bacterial profile among intervention and control neonates in the study population. The outcomes of this report were not prespecified in the original trial protocol.

### Participants

In this multi-country, randomized controlled trial, 3211 newborns 1609 in intervention and 1602 in control, with birth weight 1 to <1.8 kg irrespective of gestational age, mode of birth or single or twin gestation were enrolled at NICUs in Ghana, India, Malawi, Nigeria and Tanzania between December 2017 and January 2020. Exclusion criteria were a mother younger than 15 years of age, triplet pregnancies or more, if the mother was considered too sick to be likely to provide KMC within 72 h after birth, if the mother resided outside the defined study area, if the newborn was not able to breathe spontaneously within 60 min of birth, or had major congenital malformations. Written informed consent was taken from participants before enrolment.

### Randomisation and masking

Randomization was done by computer-generated blocks. The blocks were stratified on basis of site and birth weight (1.0 to <1.5 kg or 1.5 to <1.8 kg). The allocations were sealed in serially numbered, opaque envelopes made at WHO and sent to the sites. Research staff conducted randomization after opening numbered envelope sequentially. Twins were assigned to the same group. The nature of the intervention was such that, it could not be blinded.

### Procedures

Changes in the infrastructure and processes were necessary in the newborn intensive care units (NICUs) for intervention mother baby dyads to provide immediate kangaroo mother care. These NICUs (Mother–NICUs), which included mothers' beds and KMC chairs, were made or modified from existing NICUs. All equipment, human resource, and neonatal care protocols in the Mother–NICUs were the same as in the conventional NICUs where control babies were admitted. To ensure a patent airway while providing immediate kangaroo mother care, neonates were secured firmly to the mother's chest with a binder. All mothers and neonates were provided medical care while being in skin to skin contact without separation, as much as possible. Obstetricians provided essential postpartum care to mothers in the Mother–NICUs, just as they did for mothers in the post-natal wards. Neonates who were enrolled in the control group were transferred to the conventional NICU and their mothers were transferred to postnatal wards. Mothers visited NICU intermittently to provide expressed breast milk and short sessions of kangaroo mother care when neonates started stabilizing and were at least 24 h old.

### Minimal package of newborn care

To maintain standard of newborn care across all five study sites, it was ensured that all neonates received WHO minimum-care package for the newborn including resuscitation at birth if required, thermal care, early and frequent breastmilk feeding, infection prevention and control, and respiratory support for respiratory distress syndrome.^5^

## Outcomes

The primary outcomes for this post-hoc analysis were the incidence of sepsis in sub-groups, sepsis related mortality and bacterial profile of isolates. Definitions used in the study were adapted from Centres for Disease Control and Prevention- National Healthcare Safety Network (CDC-NHSN.).^6^

Suspected sepsis was defined as one or more of the following signs or symptoms: temperature of less than 35.5 °C or more than 38 °C, no movement or movement only on stimulation, in-drawing of the chest, and convulsions. In newborns more than 24 h of age, if newborn did not have any such sign or symptom in last 24 h and one of these signs or symptoms appeared, newborn was included as suspected sepsis. Therefore, denominator for calculating suspected sepsis rates excluded newborns who died, before reaching 48 h of age.^2^ Neonates who developed sepsis during hospital stay were included in the study.

Culture positive sepsis (laboratory-confirmed bloodstream infection) was defined as blood culture positive in a neonate with clinically suspected sepsis.

Culture-negative sepsis was defined as clinical course suggestive of sepsis, but no pathogen isolated or blood culture not done.

Sepsis related mortality was defined as percentage of randomized newborns who died of suspected sepsis.

### Blood culture methods

1–2 ml of blood (at least 1 ml) was inoculated in Paediatric blood culture bottle. The inoculated blood culture bottles were incubated at 37 °C in the BACTEC automated blood culture system for a maximum duration of seven days unless flagged positive. At 24 h, smear from the culture bottle was made if growth was suspected on receipt of the sample. Subculture was done on 5% sheep Blood Agar and MacConkey Agar. At 48 h, if positive, proceeded for identification and antibiotic sensitivity report as per the standard guidelines. If negative, subculture (s/c) in sheep Blood Agar and MacConkey Agar was repeated. On 7th day, if positive, proceeded as per standard guidelines and if negative, sample was discarded. Antimicrobial susceptibility testing (AST) of the isolates was determined as per Clinical and Laboratory Standards Institute (CLSI) guidelines. Antimicrobial resistance of the isolates was determined as per Clinical and Laboratory Standards Institute guidelines.^7^

### Definition of multi-drug resistance

The Gram-negative pathogens were considered as Multi-drug resistant based on their resistance to various antibiotic classes: extended-spectrum cephalosporins (any two of ceftazidime, ceftriaxone, or cefotaxime); carbapenems (imipenem or meropenem); aminoglycosides (any one of gentamicin, amikacin, or netilmicin); fluoroquinolones (ciprofloxacin); and piperacillin–tazobactam. We defined multidrug resistance as resistance to any three of these five antibiotic classes (adapted from Sievert and colleagues).^8^

### Data collection

Data regarding baseline characteristics of neonates, risk factors for neonatal sepsis, incidence of clinical sepsis, mortality rates and site specific sepsis rates were obtained from the database of the primary iKMC study. However, culture positivity rates, profile of bacterial isolates and their sensitivity patterns were not available in database of primary iKMC study, but was procured from clinical and laboratory records of these neonates form India, Nigeria, Malawi and Tanzania study sites, cultures were not available from the Ghana site.

### Statistical analysis

For original trial, 4200 neonates were to be enrolled to detect 20% lower mortality in the intervention group than in the control group (16.8% vs. 21.1%), with a 95% confidence level, 90% power, and a 10% loss to follow-up. The data and safety monitoring board (DSMB) performed interim analyses after 50% and 75% of the participants had been enrolled. After the second interim analysis, the data and safety monitoring board recommended to stop enrolment because of the definite benefit in neonatal survival in the intervention group.^2^ Considering the number of recruited babies, the current post-hoc analysis has an 88% power to detect a 21% reduction of sepsis (from 27.8% to 22.9%) among infants in the same birth weight category receiving immediate Kangaroo Mother Care.

Statistical analysis was done using software Stata version 15. We summarized continuous variables as means and standard deviations and categorical variables as frequencies and percentages. Differences between arms were tested using chi-square or t-tests accordingly.

For binary outcomes, adjusted risk ratios were estimated with the use of log-binomial regression modelling adjusted for clustering due to multiple births, study site, type of delivery, multiple pregnancies, mother's age at randomization, infant's sex and weight, mother's years of schooling and age, a household with toilet, and family income.

### Role of the funding source

The funder of the study had no role in study design, data collection, data analysis, data interpretation, or writing of the report. HC, SA, NM, SY and RC had access to dataset and had final responsibility for the decision to submit for publication.

## Results

**Fig. 1:**
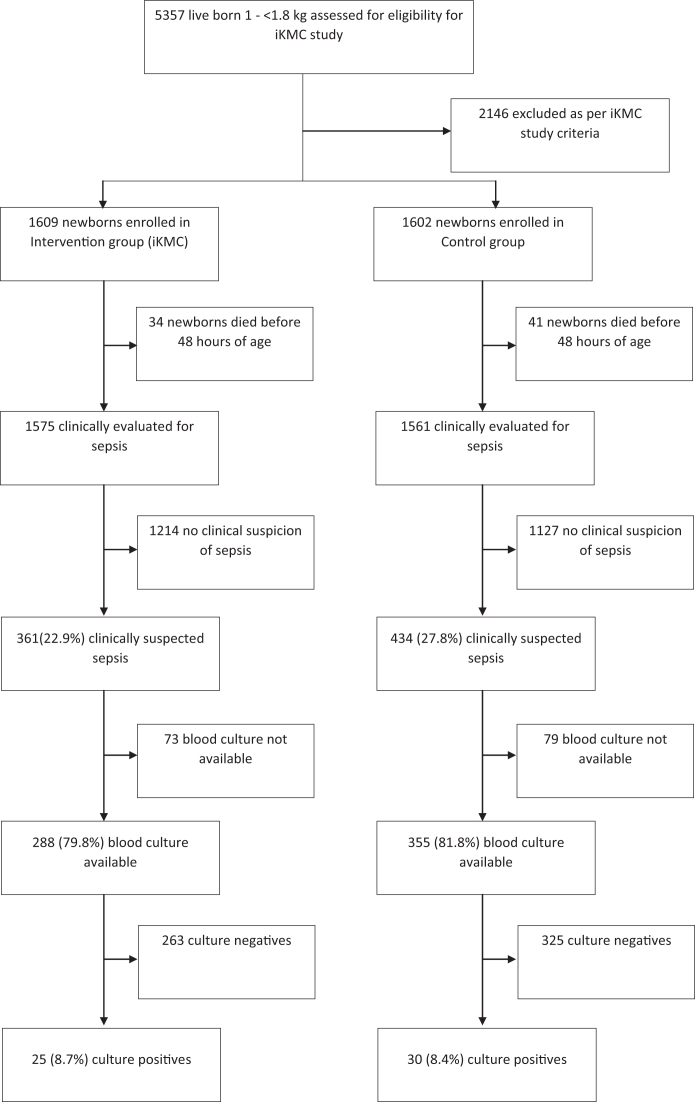
Study flow. This figure represents study flow of enrolment, clinically suspected sepsis, and culture positive sepsis.

In the intervention group 1609 newborns and in the control group 1602 newborns were enrolled in iKMC study between November 30, 2017, and January 20, 2020. 1575 newborns in the intervention group and 1561 in the control group were clinically evaluated for sepsis as 34 neonates died in intervention group and 41 died in control group before 48 h of age and could not be evaluated for sepsis (Fig. 1).

There were no statistically significant differences between groups regarding gestational age and birth weight. Mean gestational age was 32.6 (SD 3.0) gestational weeks in the intervention and 32.6 (SD2.8) in the control group, and mean birth weight was 1.5 (SD 0.2) kg in both the groups.

The maternal risk factors for neonatal sepsis; rupture of membranes more than 18 h before birth, maternal pyrexia of >38 °C and foul-smelling liquor were similar in the two groups. Neonatal care related factors which can be risk factors for neonatal sepsis; use of intravenous fluids, Continuous Positive Airway Pressure (CPAP), top feeds and duration of hospital stay were also similar in the two groups (Table 1).

**Table 1:** Characteristics of enrolled neonates and mothers.

Characteristics	Intervention	Control
**Neonatal characteristics**	N = 1609	N = 1602
Mean birth weight (in kg)	1.5 ± 0.2^a^	1.5 ± 0.2^a^
Mean gestational age (in wks.)	32.6 ± 3.0^a^	32.6 ± 2.8^a^
Male-no. (%)	752 (46.7)	748 (46.7)
Small for gestation-no. (%)	634 (39.4)	613 (38.3)
Cesarean section-no. (%)	559 (34.7)	614 (38.3)
Infants born as twins-no. (%)	430 (26.7)	430 (26.8)
Apgar score<7 at 5 min after birth-no. (%)	130 (8.1)	135 (8.4)
**Maternal characteristics**	N = 1470	N = 1474
Rupture of membrane >18 h before birth-no. (%)	212 (14.4)	204 (13.8)
Foul smelling liquor-no. (%)	24 (1.6)	16 (1.1)
Fever (>38 °C) during labor or child birth-no. (%)	5 (0.3)	6 (0.4)
**Neonatal care related variables**		
Use of Intravenous fluids-no. (%)	1129 (70.2)	1119 (69.8)
Use of CPAP-no. (%)	998 (62.0)	960 (59.9)
Use of Top feeds-no. (%)	770 (47.8)	760 (47.4)
Mean duration of hospital stay (days)	14.9 ± 0.2^a^	15.2 ± 0.2^a^

a Plus–minus values are means ± SD.

The median duration of NICU stay was 6.4 days in both groups. During the NICU stay, the median daily duration of skin to-skin contact in the intervention group was 16.9 h and that in the control group was 1.5 h. In intervention group, when mother was not available, STS was provided by another person (surrogate). Median duration of skin-to-skin contact with mother was 12.3 h per day and with surrogate 2.3 h per day.^2^

Sepsis related mortality was 37% less in intervention group than the control group; RR 0.63 (CI 0.47–0.85). Sepsis related mortality was significantly lower in iKMC group (Table 2).

**Table 2:** Incidence and mortality of neonatal sepsis.

	Intervention (n = 1575)	Control (n = 1561)	RR (95%CI)	AdjRR^a^ (95%CI)
Suspected sepsis no. (%)	361 (22.9)	434 (27.8)	0.82 (0.73–0.93)	0.82 (0.73–0.93)
Sepsis related mortality no. (%)	70/1575 (4.4%)	109/1561 (6.9%)	0.64 (0.48–0.85)	0.63 (0.47–0.85)

Suspected sepsis was defined as one or more of the following signs or symptoms: temperature of 35.5 °C or more than 38 °C, no movement or movement only on stimulation, in-drawing of the chest, and convulsions. Signs and symptoms were not reported for the first 24 h of life. After that time, the infant should have been well for at least 24 h before becoming sick. The denominator excludes infants who died before reaching 48 h of age.

a Risk ratios (RR) were adjusted for clustering due to multiple births and in accordance with study site, type of delivery, multiple pregnancies, mother's age at randomization, infant's sex and weight, mother's years of schooling and age, household with toilet, and family income.

The intervention group had similar effects across the categories of birth weight (Table 3). Suspected sepsis was 14% lower in intervention group in sub-group of neonates with birth weight 1.0-<1.5 kg; RR 0.86 (CI 0.75, 0.99). Among neonates with birthweight 1.5-<1.8 kg, suspected sepsis was reduced by 24%; RR 0.76 (CI 0.62, 0.93). In both weight categories, there was significant reduction in suspected sepsis in iKMC group. Suspected sepsis rates were lower in intervention group than in the control group across all sites (Table 3).

**Table 3:** Subgroup analyses of neonatal sepsis.

Subgroup	Intervention no. of newborns with sepsis/total no. (%)	Control no. of newborns with sepsis/total no. (%)	aRR (95%CI)
**Birth weight**			
1.0–<1.5 kg	223/678 (32.9%)	255/677 (37.7%)	0.86 (0.75, 0.99)
1.5–<1.8 kg	138/897 (15.4%)	179/884 (20.2%)	0.76 (0.62, 0.93)
**Site**			
Ghana n (%)	58/197 (29.4%)	74/198 (37.4%)	0.79 (0.59–1.05)
India n (%)	129/687 (18.8%)	145/673 (21.6%)	0.87 (0.70–1.08)
Malawi n (%)	57/206 (27.7%)	85/208 (40.9%)	0.68 (0.51–0.90)
Nigeria n (%)	16/107 (15.0%)	17/103 (16.5%)	0.91 (0.49–1.68)
Tanzania n (%)	101/378 (26.7%)	113/379 (29.8%)	0.89 (0.71–1.13)

a For sub-group analysis by birth weight, the risk ratio (RR) was adjusted according to study site and clustering owing to multiple births. For the subgroup analysis by site, adjustment was made only for clustering owing to multiple births.

Culture positivity was 8.7% (25/288) in the intervention group and 8.4% (30/355) in the control group. Profile of bacterial isolates was different in the intervention and the control group. Intervention group had fewer Gram-negative isolates (n = 9) than Gram positive isolates (n = 16). While control group had more Gram-negative isolates (n = 18) than Gram positive (n = 12) (Table 4).

**Table 4:** Profile of bacterial isolates.

	Intervention (n = 25)	Control (n = 30)
**Gram Negative**	**9 (36%)**	**18 (60%)**
Klebsiella spp.	5	8
Acinetobacter spp.	2	1
Pseudomonas spp.	1	2
Enterobacter spp.	1	5
Proteus spp.	0	1
*Escherichia coli*	0	1
**Gram Positive**	**16 (64%)**	**12 (40%)**
*Staphylococcus aureus*	9	8
Coagulase negative staphylococci	3	2
Enterococcus spp.	4	0
Streptococcus spp	0	2

Among Gram-negative organisms, Klebsiella spp. and among Gram-positive, Methicillin Resistant *Staphylococcus aureus* were predominant in both intervention and control groups. Five multi-drug resistant Gram-negative isolates were cultured, out of which one was in the intervention group and four were in the control group.

## Discussion

Continuous presence of mothers and surrogates in the NICU to provide iKMC does not increase neonatal sepsis but may instead reduce it.

Previous studies have also shown reduction in rates of neonatal sepsis by use of KMC in clinically stable newborns.^1^^,^^9^^,^^10^ As compared with the studies of KMC in clinically stable newborns in the Cochrane review,^1^ the present iKMC study achieved a much higher median duration of the intervention i.e., approximately 17 h of skin-to-skin contact per day.^2^

There are several possible mechanisms by which iKMC might reduce the incidence of neonatal sepsis. Since immediate KMC was started within 2 h of birth and then continuous KMC in MNCU was provided, neonates in the intervention group were likely to be colonized by mother's protective microbiota rather than the NICU environment and hospital staff. Studies have shown that skin-to-skin care in preterm babies leads to a distinct microbial pattern in the oral cavity.^11^

Another protective mechanism by which KMC may reduce neonatal sepsis, is that the neonates in the iKMC group received earlier and more frequent breastmilk feeding due to continuous presence of mother with the baby in MNCU.^2^

In the MNCU, there is reduced risk of cross infection since each mother cares only for her newborn while each nurse cares for 8–10 neonates in same shift in the conventional NICU.

In the present iKMC study, the sepsis related mortality was 37% lower in the intervention group than the control group. There are several explanations for this finding. Constant monitoring of the neonate by the mother for danger signs might have led to early suspicion of sepsis and its complications, leading to her raising the alarm for timely management, resulting in reduced mortality by sepsis in intervention group. Studies have also shown that oxidative stress biomarkers are reduced in neonates who receive KMC and therefore, reduction of stress in neonates related to mother–newborn separation may have contributed to reduced mortality due to sepsis.^12^

In the present iKMC study, profile of bacterial isolates was different in the intervention and control group. This may be the effect of colonization with maternal microbiome in the iKMC group. The study which evaluated the microbial pattern in the oral cavity in preterm neonates with or without Skin-to-Skin contact (SSC) found that SSC was associated with increased colonization with Streptococcus, while non-SSC was associated with greater colonization with Corynebacterium and Pseudomonas in newborns ≤32 weeks corrected gestational age. SSC was associated with shorter hospitalization.^11^

There were much fewer Gram-negative isolates in the intervention group, while Gram-positive isolates were more frequent in the intervention group. This may also explain reduced sepsis related mortality in intervention group as studies have shown Gram-positive neonatal sepsis has a lower mortality than Gram-negative sepsis.^13^ Five multi-drug resistant Gram-negative isolates were cultured out of which one was in the intervention group and four were in the control group. Although the number of multi-drug resistant isolates were very low, it was less prevalent in the intervention group which may also have affected sepsis related mortality.

Research from high-income countries would help to determine whether these reduced rates of sepsis and sepsis-related mortality in low- and middle-income countries are also applicable to settings in which sepsis related mortality is low and level of intensive neonatal monitoring high. One such study is already underway from Scandinavia.^14^

There are some limitations of these findings related to the effect of iKMC on neonatal sepsis. Although it was an RCT with no selection bias, but intervention could not be blinded, so there is limitation of possibly biasing the estimates. In one of the study sites, blood culture and drug sensitivity were not available. The number of culture-positive isolates was too small to analyse occurrence of individual microorganisms among intervention and control groups. Possible reasons for low isolation might be starting antibiotics before taking blood culture and non-adherence to strict protocol of blood culture collection including inadequate sample and improper transport. The number of multi-drug resistant isolates were too small to compare statistically between the two groups.

Studies are needed to correlate bacterial isolates from cases of neonatal sepsis in iKMC and conventional care with pattern of mothers’ microbiome, to learn about the possible effects of maternal microbiome on neonatal sepsis when practising iKMC.

In conclusion, contrary to a widespread concern, continuous presence of mothers and surrogates in the NICU to provide iKMC does not increase neonatal sepsis but rather reduces it. Profile of bacterial isolates is different in iKMC and control group, and sepsis related mortality is lower in the iKMC group.

## Contributors

All authors contributed to study design and methods. SA and HC did literature review and drafted the manuscript. SA, HC, NM, SY and RC directly accessed and verified the underlying data reported in the manuscript. SA and HC are from academic team. All authors contributed in interpretation of data, critically revised the manuscript and agreed on the final version.

## Data sharing statement

Data will not be publicly available. Data can be provided on request to the corresponding author with permission from WHO Geneva and the involved hospitals.

## Declaration of interests

All authors declare no competing interests.
